# Stem cells under the influence of alcohol: effects of ethanol consumption on stem/progenitor cells

**DOI:** 10.1007/s00018-018-2931-8

**Published:** 2018-10-10

**Authors:** Giuliana Di Rocco, Silvia Baldari, Giovambattista Pani, Gabriele Toietta

**Affiliations:** 1 Department of Research, Advanced Diagnostic, and Technological Innovation, Translational Research Area, IRCCS Regina Elena National Cancer Institute, Via E. Chianesi 53, 00144 Rome, Italy; 2 Institute of General Pathology, Laboratory of Cell Signaling, Catholic University Medical School, Largo F. Vito 1, 00168 Rome, Italy

**Keywords:** Alcoholic beverages, Mesenchymal stromal cells, Alcohol-related disorders, Fetal alcohol spectrum disorders, Alcohol dehydrogenase, Acetaldehyde

## Abstract

Stem cells drive embryonic and fetal development. In several adult tissues, they retain the ability to self-renew and differentiate into a variety of specialized cells, thus contributing to tissue homeostasis and repair throughout life span. Alcohol consumption is associated with an increased risk for several diseases and conditions. Growing and developing tissues are particularly vulnerable to alcohol’s influence, suggesting that stem- and progenitor-cell function could be affected. Accordingly, recent studies have revealed the possible relevance of alcohol exposure in impairing stem-cell properties, consequently affecting organ development and injury response in different tissues. Here, we review the main studies describing the effects of alcohol on different types of progenitor/stem cells including neuronal, hepatic, intestinal and adventitial progenitor cells, bone-marrow-derived stromal cell, dental pulp, embryonic and hematopoietic stem cells, and tumor-initiating cells. A better understanding of the nature of the cellular damage induced by chronic and episodic heavy (binge) drinking is critical for the improvement of current therapeutic strategies designed to treat patients suffering from alcohol-related disorders.

## Introduction

**Fig. 1:**
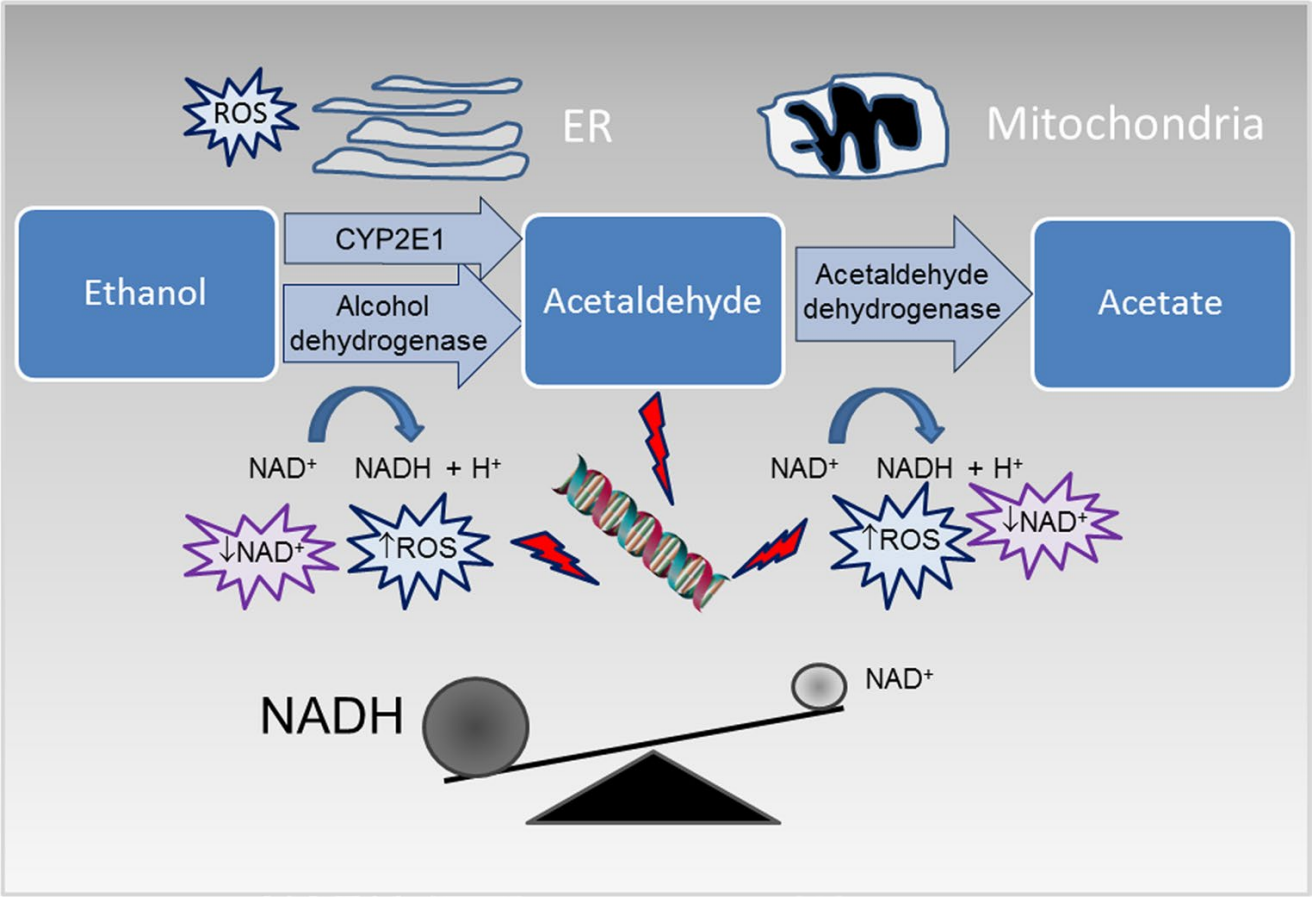
Main pathways of alcohol oxidative metabolism. Ethanol is converted into acetaldehyde by the cytosolic enzyme alcohol dehydrogenase and by the microsomal cytochrome P450 2E1 (CYP2E1); acetaldehyde is then oxidized into acetate by the mitochondrial enzyme aldehyde dehydrogenase 2. Alcohol metabolism increases the ratio of the reduced nicotinamide adenine dinucleotide (NADH) to the oxidized nicotinamide adenine dinucleotide (NAD^+^) and promotes reactive oxygen species (ROS) production

According to the World Health Organization, approximately half of the global adult population consumes alcoholic beverages; nevertheless, alcohol consumption represents one of the most important risk factor for public health and the third leading cause of premature death [1]. Alcohol use is associated with chronic and acute diseases including several types of cancers, cardiovascular diseases, diabetes, pneumonia, immunologic alterations, liver cirrhosis, and reduced healing after traumatic injury [2]. Moreover, alcohol exposure during prenatal gestation determines a wide array of harmful effects on the developing fetus, collectively indicated as fetal alcohol spectrum disorders (FASD). Whether moderate alcohol consumption is potentially preventative against cardiovascular diseases [3] is an object of ongoing scientific debate [4]. The quantity, type, and quality of the alcoholic beverages and the pattern of drinking habits can modify the effects of alcohol misuse. Moreover, several other factors, such as genetic background, gender, age, ethnicity, diet, hormone status, as well as interactions with additional risk factors such as smoking and obesity, can have an impact on modulating alcohol response [5]. In addition, the rate of alcohol metabolism contributes to the development of alcohol-associated diseases. Ingested alcohol is rapidly absorbed throughout the gastrointestinal tract into the bloodstream, and then, the majority (more than 90%) is metabolized in the liver. The most important hepatic ethanol metabolic pathway involves the oxidation by the enzymatic activity of alcohol dehydrogenase (ADH), which converts alcohol to acetaldehyde, and acetaldehyde dehydrogenase (ALDH), which converts acetaldehyde to acetate (Fig. 1). As an ultimate step, acetate is further metabolized to carbon dioxide and water before being eliminated from the body. The kinetic of the process may be altered in the presence of different polymorphic variants of these enzymes [6]. Overall, alcohol metabolism results in: production of acetaldehyde, which is a known carcinogen; generation of reactive oxygen species (ROS), contributing to oxidative stress; production of acetate, which affects several metabolic processes; and in a significant alteration of the cellular redox balance, in particular through a change of the ratio of the oxidized and reduced forms of nicotinamide adenine dinucleotide (NAD^+^/NADH) [7].

Ethanol has an impact on virtually all tissues, and some effects can persist long after stopping alcohol intake. Growing and developing tissues are remarkably susceptible to alcohol due to incomplete antioxidant protection. In fact, epidemiologic studies have provided evidence that drinking during adolescence can be dangerous for brain development, possibly inducing irreversible damage. Most importantly, alcohol exposure in pregnancy affects embryo development and can cause permanent birth defects. Similarly, early postnatal alcohol exposure might result in immunological and neurological disorders in later life. Accordingly, teen and prenatal alcohol drinking are serious social and health problems with increasing incidence. While the fact that acute and chronic alcohol abuse severely damages tissue and organ functions is well-established, only recently the role of ethanol exposure in deteriorating stem-cell properties has been investigated. Different organs of an individual may have different sensitivities to alcohol. The hypothesis that ethanol has nefarious consequences on stem cells in different tissues is consistent with alcohol’s widespread, long-term effects observed in the developing and adult human organisms.

**Fig. 2:**
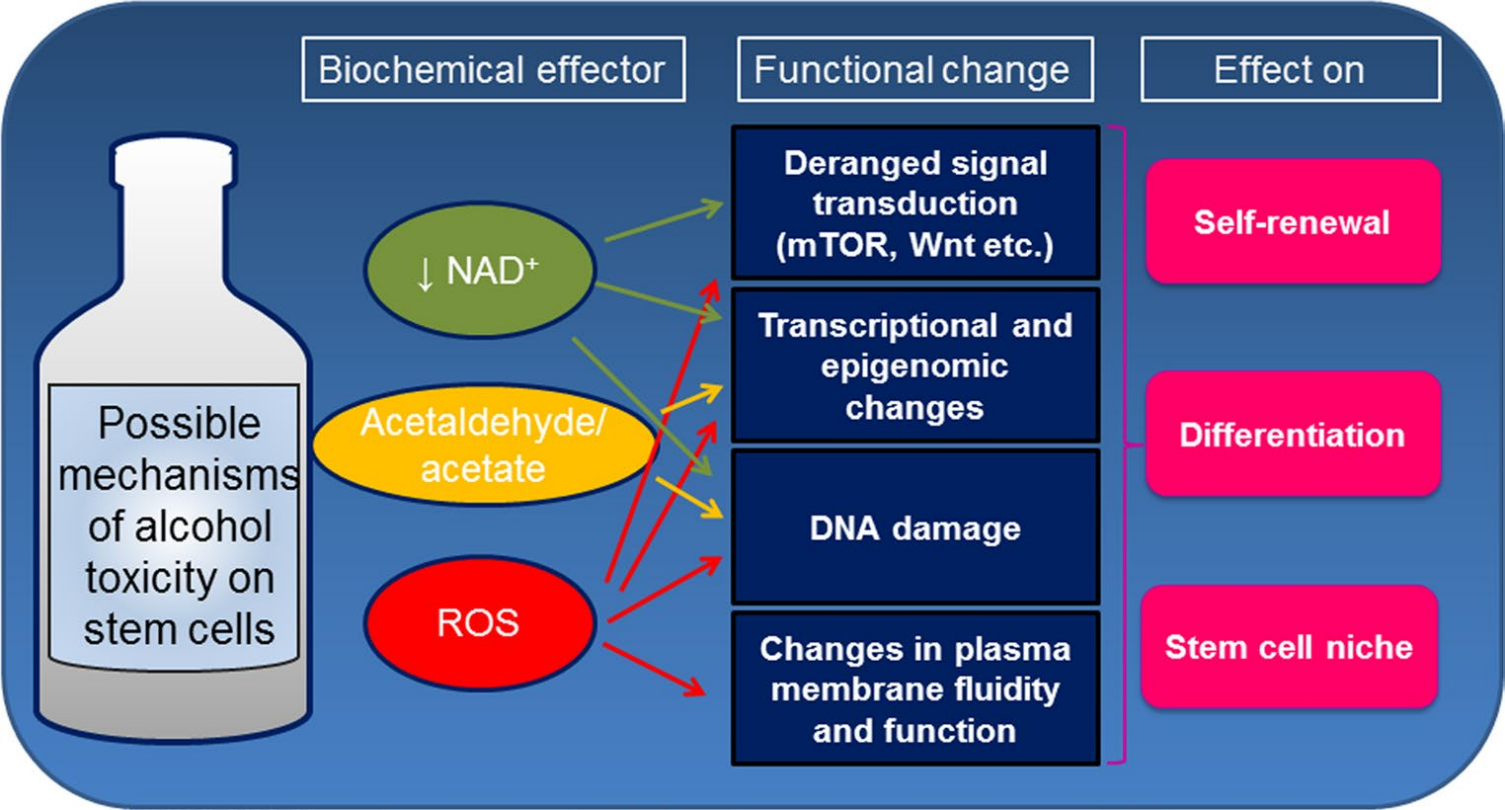
Possible mechanisms of alcohol toxicity on stem cell biology

Evidence of direct toxicity of ethanol and/or its metabolites on stem and progenitor cells in different tissues offers a new perspective on the biological consequences of alcohol intake (Fig. 2). Accordingly, due to the profound perturbation of alcohol on stem-cell biology in the developing fetus, Mahnke et al. have provocatively proposed to classify FASD as a “stem cellopathy” [8]. However, the exact mechanisms causing ethanol deleterious effects remain to be completely investigated. Alcohol susceptibility may depend on the target cell and may be influenced by the dose and duration of exposure. Adult stem cells reside in a quiescent state in specialized niches within multiple different tissues. In response to an injury stem cells are able to exit quiescence, renew themselves and differentiate into different cell types [9]. The maintenance of quiescence for a prolonged period of time is functional to preserve stem-cell proliferative potential and genomic integrity throughout the lifetime of the organism. On the other hand, long-lived stem cells are continuously exposed to endogenous and exogenous genotoxic agents, leading to accumulation of DNA damage. Accumulating evidences, suggest that alcohol may impact key aspect of stem cell biology, such as the function of the niche, maintenance of potency and differentiation. In the following sections, we review the main studies describing the effects of alcohol on different types of stem cells (Table 1).

**Table 1:** Effects of alcohol on stem cells

Cell type	Observed changes	Main references
Neural progenitors cells	Reduced proliferation; effects on cell fate determination	[10, 16, 17, 20]
Hepatic stem cells	Reduced proliferation; promotion of mesenchymal transition	[43]
Intestinal stem cells	Decreased the expression of stem cell markers,	[46]
BM-derived MSC	Reduced osteogenic differentiation; adipogenic effect; induction of senescence	[34, 52, 57, 59]
Dental pulp stem cells	Dysregulation of odontogenic/osteogenic differentiation.	[58, 63, 64]
Adventitial progenitor cells	Decreased proliferation; reduced myogenic differentiation	[69]
Embryonic stem cells	Inhibition of differentiation; apoptosis induction	[78, 86]
Hematopietic progenitors	Effects on cell fate determination; DNA damage	[91–93, 97]
Cancer stem cells	Phenotypic changes; microenvironmental alterations.	[105, 108]

*BM* bone marrow, *MSC* mesenchymal stromal cells

## Effects of alcohol on different types of stem cells

### Neural stem cells

The devastating effects of fetal exposure to ethanol on brain development and function in the context of FASD conceivably reflect major consequences of alcohol on early neuronal maturation and by extension on proliferation, differentiation and survival of embryonic neural stem and progenitor cells (NSC) [8, 10]. Furthermore, ethanol can also impact on adult NSC activity in some brain areas, primarily in the subgranular zone of the hippocampus, where neurogenesis persists throughout the entire lifetime [11], or at least until early postnatal life [12]. Adult neurogenesis has been documented in nearly all animal models investigated so far, including rodents and primates, supporting a limited degree of cell renewal within brain structures which has been estimated in 1.7% annual turnover in the human hippocampus [13]. Adult neurogenesis contributes to higher cognitive phenomena, from pattern discrimination to memory consolidation/extinction and mood changes [14]. Thus, deranged adult neurogenesis may contribute to the neurologic decline and neurodegenerative changes observed in chronic alcoholism [15–17]. Accordingly, studies in adolescent and adult rodents under a binge ethanol exposure have shown a consistent reduction in neural progenitors proliferation and survival, as assessed by bromodeoxyuridine (BrdU) labeling at both 1 and 28 days after treatment [18, 19]. A similar, albeit transient (maximum at 3 days with recovery at 30 days), reduction of dentate gyrus (DG) progenitors has been described in rats subjected to chronic, voluntary ethanol assumption in the drinking water [20]. Conversely, neurogenesis increases in rats during chronic abstinence from alcohol [21], a phenomenon possibly related to the return of human cognitive function and brain volume associated with recovery from addiction. Of note, brain damage and epilepsy, two conditions experimentally associated with enhanced hippocampal neurogenesis [22], are also major consequences of ethanol abuse on human brain.

Interestingly, compromised adult neurogenesis may also be implicated in the long-term neural consequences of fetal exposure to ethanol. In particular, adult neurogenesis appears preserved [23] or even increased in adult rodents prenatally exposed to ethanol [24], with higher number of immature neurons in DG, a possible compensatory mechanism to alcohol-induced neuronal loss. In contrast, a decrease in hippocampal neurogenesis has been specifically detected in aged rats exposed to the same experimental paradigm [25]. While the mechanisms underlying these age-specific effect of fetal ethanol exposure on adult neurogenesis are still ill–defined, alcohol-induced epigenetic changes involving the neural stem cell pool [26–28], as well as non-cell autonomous changes affecting neural stem cell niche [29] have been considered.

Molecular cascades connecting exposure to ethanol with the defects in NSC proliferation, differentiation and survival that overall result in impaired developmental and adult neurogenesis are still elusive. A reduction of brain-derived neurotrophic factor (BDNF), a major neurogenic neurotrophin, has been reported in plasma of alcohol-addicted patients [30]. Consistently, BDNF as well as insulin-like growth factor-1 have been shown to ameliorate the inhibition of rat embryonic NSC differentiation induced by ethanol in vitro (20–100 mM) [31]. Moreover, physical exercise, that reportedly increases hippocampal BDNF, was able to attenuate the long-lasting hippocampal neurogenic deficits in a rat model of FASD [32]. Downstream of neurotrophin receptors, the mammalian target of rapamycin (mTOR) and its effectors have been recognized key roles in the modulation of NSC functions [33], but their specific involvement in ethanol effects on neurogenesis have been little investigated. Ethanol metabolism promotes the microsomal and mitochondrial generation of ROS in several cell models including stem/progenitor cells [34]; on the other hand, increased levels of oxygen species inhibit mTOR activity and promote autophagy in neurons via a peroxisome–tuberous sclerosis complex 2 circuitry [35]. Thus, ethanol-induced proliferative defects of NSC involve impaired signaling capacity along the mTOR cascade. With this respect, it is of note that ethanol activates autophagy in the developing brain, and that autophagic preconditioning alleviates ethanol-induced ROS and neuronal damage [36].

### Liver stem/progenitor cells

Alcohol consumption causes a wide spectrum of hepatic disorders ranging from mild fatty liver (steatosis) to more severe steatohepatitis, progressive fibrosis, cirrhosis and hepatocellular carcinoma, which are collectively recognized as alcoholic liver disease (ALD) [37]. The high prevalence of ALD has a deep impact on public health; nonetheless, the pathogenesis of the disease remains poorly understood. Alcohol-induced alteration of the intestinal barrier integrity results in the translocation of bacteria, endotoxins and peptidoglycans into the systemic and portal circulation, potentially promoting liver inflammation and injury; this hepatic response to intestinal inflammation is known as the gut-liver axis [38]. Liver responds to an insult inducing mature hepatocytes proliferation; therefore, physiologic hepatic regeneration occurs with minimal involvement from liver stem cells. Hepatic ethanol metabolism increases the production of reactive oxygen species, which enhance oxidative stress and inhibit hepatocyte proliferation [39]. Accordingly, both acute and chronic ethanol exposure impair liver regeneration [40]. Reduction of the capacity of the hepatocytes to replace damaged tissue parallels the induction of proliferation of the hepatic stem cells located in the Canals of Hering. This phenomenon, referred as ductular reaction, has been linked to alcohol hepatitis progression and severity [41], increased pro-fibrogenic response and tumor development [42].

In addition, in vitro studies suggest an hepatotoxic effect of alcohol exposure on hepatic stem cells [43]. Primary and immortalized human liver stem cells exposed in vitro to ethanol (tested dose range 25–100 mM, for 72 h) show reduced proliferation rate, decreased differentiation towards a hepatocyte phenotype and induction of mesenchymal transition [43]. Taken together, these results suggest that the effect of alcohol consumption on hepatic stem cells might be multifaceted: on the one hand, alcohol and/or its metabolites might exert their toxic effects directly on hepatocytes, damaging their function and limiting their proliferative capacity; on the other hand via the gut-liver axis alcohol might stimulate inflammatory signals leading to extensive proliferation of liver stem cells, which in turn promotes fibrosis at expense of hepatic regeneration.

### Intestinal stem cells

Alcohol ingestion causes alteration of the intestinal microbiota composition and the disruption of intestinal barriers promoting local and systemic inflammation and tissue injury [44]. Intestinal epithelium cells’ turnover is rapid, supported by continuous replication of undifferentiated intestinal stem cells located within the crypts, which generate cells undergoing subsequent differentiation during the migration along the crypt-villus axis [45]. Intestinal damage persists long after alcohol consumption, suggesting that the damage may involve intestinal stem cells [46]. In vivo studies on a mouse model of alcohol chronic consumption and ex vivo organoid culture, demonstrated that alcohol exposure impairs intestinal stem-cell homeostasis, reducing their proliferation rate via deregulation of the β-catenin pathway [46]. Interestingly, alcohol exposure also significantly modifies the expression of intestinal stem-cell markers, including the leucine-rich repeat-containing G-protein coupled receptor 5 (Lgr5), which is known to be required to maintain the adult intestine epithelial cell proliferation [47]. Therefore, by limiting mucosal repair capacity alcohol-induced damage of intestinal stem cells may contribute to the alteration of intestinal barriers observed in acute and chronic drinkers [48].

### Bone-marrow-derived mesenchymal stromal cells

Alcohol abuse is associated with impaired bone homeostasis leading to pathologic remodeling and impaired healing of bone fractures [49, 50]. Bone-marrow-derived stromal/stem cells are involved in the process of physiological maintenance of bones. Alcohol imbalances the process of bone remodeling by impairing bone-marrow stromal cell osteoblastic differentiation and promoting adipogenesis, therefore, contributing to bone loss. Mechanistically, alcohol and its main metabolite acetaldehyde affect the redox state of mesenchymal stem cell niche determining the downregulation of the Wnt/β-catenin signaling pathway, which directs osteoblast and chondrocyte cells differentiation from a common mesenchymal stem cell precursor [34, 51–53]. Deregulation of Wnt signaling due to alcohol exposure before bone injury persists up to 2 week post-fracture. Consequently, differentiation of mesenchymal precursors into bone and cartilage-forming cells is impaired, inhibiting the formation of the external callus [54].

A crosstalk between the Wnt/β-catenin and the mammalian target of rapamycin (mTOR) signaling has been identified in maintenance of bone-marrow-derived [55] and epithelial stem cells [56]. Alcohol impairs bone-marrow-derived mesenchymal cells osteogenic differentiation by activation of the mTOR pathway [57], consistently with a similar effect observed on odontoblastic differentiation of dental pulp cells [58]. Moreover, in vitro exposure to 50 mM ethanol promotes a shift in bone-marrow stromal cell differentiation towards the adipocyte lineage via up-regulation of the peroxisome proliferator-activated receptor (PPAR)γ2 signaling [59].

Interestingly, the effects of chronic alcohol intoxication on bone remodeling are not permanent and can be improved by abstinence [60]. A better understanding of the molecular pathways involved in the development of alcohol-induced bone disease may help the identification of targets for pharmacological therapies. For instance, treatment with mTOR inhibitors such as rapamycin might be useful for reducing the impact of alcohol-induced bone disease [57].

### Dental pulp stem cells

Alcohol abuse, in association with other co-morbidity factors such as poor nutrition, vitamin deficiencies, and smoking, may lead to periodontal disease [61]. Dental mesenchymal stromal cells contribute to tooth homeostasis and repair of damaged dentine. These cells are present in adult dental pulp and can differentiate into osteogenic and odontogenic lineages depositing mineral matrix and trans-differentiate into neuronal cells [62]. Acute exposure of cultured human dental pulp stem cells to increasing concentration of ethanol (tested dose range 1–50 mM, for 24–48 h) results in transcriptional changes [63] and in DNA methylation pattern alterations [64]. Specifically, ethanol exposure reduces the expression profile of genes involved in the mineralization process, such as alkaline phosphatase, bone morphogenic protein 2 and 4, osteocalcin and osteopontin. The mammalian target of rapamycin (mTOR) plays a pivotal role in regulating proliferation and cell fate of mesenchymal stromal cells [65]. Accordingly, ethanol-mediated activation of the mTOR signaling pathway reduces odontoblastic differentiation of dental pulp cells [58]. Moreover, ethanol exposure leads to significant dysregulation of the lysine specific histone demethylase 6B (KDM6B) that epigenetically regulates odontogenic differentiation of dental mesenchymal stromal cells [66]. Therefore, epigenetic changes leading to altered gene expression profile may represent one of the molecular mechanisms underlying the effect on dental mesenchymal stromal cells contributing to ethanol-associated periodontal disease.

### Adventitial progenitor cells

The vessel wall contains a heterogeneous population of stem cells, referred as adventitial stem/progenitor cells, able to differentiate into vascular and non-vascular cells [67]. These stem cells are involved in the process of vascular remodeling in response to arterial injury and disease. Alcohol consumption has a complex effect on the cardiovascular system; several evidences indicate that low drinking is associated with a beneficial effect on the pathophysiologic mechanisms involved in most cardiovascular disorders [3], while alcohol abuse has been linked to an increased risk [68]. Interestingly, Fitzpatrick et al. has recently demonstrated on a murine model that ethanol exposure equivalent to two drinks daily reduces stem cell antigen (Sca)-1 positive adventitial progenitor-cell proliferation via inhibition of the sonic hedgehog pathway [69]. Therefore, moderate drinking, acting on vascular stem cells, may contribute in attenuating pathologic arterial remodeling [70]. However, possible positives effect of moderate drinking on the cardiovascular system might be outweighed by the harmful impact of alcohol on other tissues and organs, implying that “no level of alcohol consumption improves health” [4, 71].

### Embryonic and induced pluripotent stem cells

Embryonic stem cells (ESCs), which are derived from the inner cell mass of blastocysts, and induced pluripotent stem cells (iPSCs), which are adult cells genetically reprogrammed to an embryonic stem cell-like state, represent attractive in vitro models for the study of human developmental studies [72, 73]. Alcohol exposure alters the gene expression and methylation profiles in ESCs [74]. Human embryonic cells can model the ethanol-mediated developmental toxicity [75, 76] and different lines of evidence consistently show that alcohol exposure deregulate the differentiation properties of ESCs derived from both murine and primate sources. In particular, human ESCs hepatic differentiation is impaired by alcohol-mediated inhibition (tested dose range 25–100 mM) of the mitogen-activated protein kinase/extracellular-signal-regulated kinase (MAPK/ERK) and WNT signaling pathways [77]. Similarly, ethanol (tested dose range 17.1–51.4 mM) suppresses cardiac differentiation of murine ESCs by inhibiting Wnt signaling [78, 79]. In addition, neurological differentiation of ESCs is compromised by ethanol exposure (tested dose range 25–100 mM) via alteration of the equilibrium of the expression of Sox2, Oct4, and Nanog [80, 81]. In addition, alcohol exposure induces gene expression changes in hESC-derived cortical neurons, including a significant up-regulation of *N*-methyl-d-aspartate (NMDA) receptor subunit gene expression [82]. Alcohol-mediated changes in NMDA receptor function was also assessed in iPS-derived neural cells [83], indicating that iPSCs may offer a novel approach for better understanding the molecular mechanisms of alcohol use disorders [73, 84]. Exposure of iPSc to alcohol (100 mM) was also demonstrated to induce apoptosis and impair hepatic differentiation [85].

Collectively these data suggest that ESCs are more sensitive to ethanol than differentiated cells [75, 86] providing a possible mechanism by which prenatal alcohol exposure exerts its toxic effect on different target organs. It should be considered that during physiological embryonic development, ESCs are present for a limited period, so the effect of alcohol on development may be directly due to the effect on ESCs but most probably also on their progeny at different stages of differentiation [86].

### Hematopoietic stem cells

Alcoholic patients may experience anemia, bleeding disorders, and compromised immune response [87]. Alterations in the hematologic profile have been also observed in short-term moderate alcohol drinkers. Hematopoiesis is a coordinated process of cell proliferation, self-renewal, and differentiation from a small population of bone-marrow (BM) hematopoietic stem cells (HSCs) to lineage-specific terminally differentiated cells. Nutritional deficiencies associated with alcoholism may negatively influence the production and function of various blood cells. On the other hand, direct effects of alcohol toxicity may target the BM, leading to the generation of abnormal blood cell precursors that cannot mature into functional cells [87]. In line with this hypothesis, the formation of vacuoles, which interfere with cells’ functionality, has been observed in red and white blood cells precursors [88]. In physiological conditions only a small fraction of HSCs enter cell cycling for self-renewal and/or proliferation, while the majority are maintained in the quiescent state. Prolonged quiescence exposes HSCs to endogenous and exogenous genotoxic agents promoting accumulation of a severe mutation burden with harmful effects, possibly leading to anemia, increased risk of developing cancer and cellular ageing [89]. Alcohol as such is not mutagenic but its metabolism produces ROS and acetaldehyde, that is mutagenic and carcinogenic via induction of DNA damage, interference with DNA replication and formation of DNA adducts [90]. It has been elegantly demonstrated that endogenous aldehydes, such as the ones produced during ethanol metabolism, are genotoxic to cells of the hematopoietic system. In the absence of the protective role of the Fanconi anemia DNA repair pathway and the activity of an isoform of aldehyde dehydrogenase (ALDH), the genotoxic effect of acetaldehyde results in a severe depletion of the HSC pool [91]. The same authors extended their findings establishing a key role of p53 in response to aldehyde-induced DNA damage to promote HSCs loss [92]. Collectively, the seminal work performed by Garaycoechea and collaborators improved our knowledge on the impact of alcohol consumption on human health, providing the molecular link between alcohol exposure and HSC damage. Consistently, formaldehyde produced by the metabolism of methanol, which may be also present in alcoholic beverages, exerts a genotoxic effect on HSCs [93].

Alcoholic patients may experience also reduced granulocyte levels and show weakened host immune defense against bacterial infection [94]. It has been demonstrated that alcohol administration alters the molecular cues needed for HSC activation and production of granulocytes, compromising the immune response against pathogens [95, 96]. In addition, in utero alcohol exposure affects the transcriptional regulation of B cell development from hematopoietic progenitors [97].

Hematopoiesis also involves the interaction of the developing cells with the BM stroma, which provides the microenvironment suitable for cells differentiation. Besides damaging HSC directly ethanol could affect the BM progenitor cells also in an indirect fashion by altering their microenvironment. Alcohol has a detrimental effect also on BM stromal cells, which has been associated with the reduction of bone mass and decreased bone formation observed in alcoholics [52]. In addition, alcohol-mediated liver injury promotes mobilization of BM-derived CD34^+^ stem cells which mainly contain hematopoietic and endothelial progenitor cells. Hepatic recruitment of CD34^+^ stem cells correlates with liver fibrosis [98]. Therefore, alcohol promotes dysfunctional hematopoiesis acting on bone-marrow cells at various stages of lineage commitment.

### Cancer stem cells

Alcohol beverages consumption is classified as carcinogenic by the World Health Organization. In particular, acetaldehyde, the first compound of alcohol metabolism, is a known carcinogen and a key generator of free radicals which can promote cancer development through multiple mechanisms [99]. Ethanol exposure results in reduction of inflammatory mediators, with immunologic alterations leading to reduced healing after traumatic injury, increased susceptibility to infection and tumor formation in the upper aero-digestive tract, colorectum, liver and breast [100]. It has been estimated that approximately 6% of all cancer deaths are attributable to alcohol, with a loss of about 19 years of potential life for each victim [101]. Alcohol may influence cancer incidence by modulating the initiation, promotion, progression or metastasis of tumors. Suggested mechanisms by which alcohol consumption increases the risk of cancer include: a tumorigenic effect of the ethanol metabolite acetaldehyde; increased levels of estrogen, which can cause breast cancer; liver cirrhosis leading to hepatocellular carcinoma; increased level of reactive oxygen species, which may damage DNA; inhibition of DNA methylation; higher solubility of tobacco carcinogenic chemicals in the mouth and throat; adverse effect on folate metabolism, with increased risk of colorectal cancer; direct or indirect alteration of oncogenic or regulatory pathways [100]. The exact cellular and molecular processes and their contribution to alcohol-associated carcinogenesis are not fully elucidated [99]. Interestingly, in a mouse model of ethanol intoxication, loss of the tumor suppressor p53 promoted alcohol-induced dysplastic changes while abrogating cell death, suggesting that p53-dependent apoptosis restrains the tumorigenic effect of ethanol on liver cells [102].

According to the cancer stem cell hypothesis, only a small subgroup of tumorigenic stem-like cells within a highly heterogeneous cancerous cell population initiates and drives tumor growth [103]. These cells, referred also as tumor-initiating cells, have characteristics similar to normal adult stem cells having both the capacity to self-renew and to differentiate into multiple cell types. Studies aimed at the identification and analysis of cancer stem cells are limited by the lack of specific cancer stem-cell markers [104]. Nonetheless, it has been proposed that alcohol may impinge on cancer stem-cell properties [105, 106]. The molecular mechanism by which ethanol metabolism contributes to tumorigenesis may be associated to the production of ROS which play a pivotal role in cancer stem-cell maintenance and differentiation [105, 107]. Indeed, alcohol promotes migration, invasion and propagation of breast cancer stem cell population via the ErbB2/p38γ MAPK axis [108]. Alcohol-mediated induction of hepatic cancer stem cells via the activation of the toll-like receptor 4 (TLR4)-Nanog pathway has also been described [109]. Moreover, a possible role of alcohol in promoting expansion of a cancer stem cell-like population in oro-esophageal squamous cell carcinoma has been recently postulated [110]. Cancer stem-cell-directed therapeutic approaches might represent an important strategy to improve cancer therapy. Therefore, improved knowledge of how and to which extent cancer stem cell biology is modified by alcohol may prove valuable for reducing the risk of ethanol-induced tumors.

## Alcohol toxicity on stem cells: in search for molecular mechanisms

Despite intensive research, the mechanisms leading to alcohol toxicity are still largely unclear. In the following sections, we review the main putative mechanisms by which alcohol might exert negative effects on stem cells (Fig. 2).

### Increased production of reactive oxygen species

Endogenous reactive metabolites, such as ROS and aldehydes produced during ethanol metabolism, are key players in induction of mutagenesis and ageing. In particular, high cellular levels of ROS can be detrimental, leading to lipid peroxidation and DNA damage. Alcohol intake can lead to ROS production by several mechanisms including ethanol and acetaldehyde oxidation by the ADH and ALDH, cytochrome P450 2E1 (CYP2E1) induction, raise in the cellular amount of free iron, conversion of xanthine dehydrogenase to xanthine oxidase [111]. Increased ROS production, impaired levels of antioxidant defense and consequent induction of oxidative stress have a causative role in FASD [112]. On the other hand, ROS are implicated in the long-term maintenance of different stem cell pools, regulating stem cell renewal, commitment and differentiation [113]. The overall amount of ROS in stem cells is determined by the balance between production and scavenging systems finely tuned by complex regulatory mechanisms. Therefore, alcohol alteration of the ROS balance may lead to functional stem cell decline which may affect organ and tissue development and regeneration [114].

### Role of the mammalian target of rapamycin

The mTOR–mitochondria–ROS axis plays a pivotal role in regulating stem-cell quiescence and self-renewal [115]. Ethanol activation of mTOR has been described in different stem cells leading to impaired differentiation [57, 58]. Moreover, in a mouse model of tuberous sclerosis, deregulated mTOR signaling led to enhanced generation of subventricular zone neural progeny, followed by premature differentiation and impaired maturation during both embryonic and postnatal development [116], a phenotype somehow reminiscent of neurogenic changes described in young mice following prenatal exposure to ethanol [24]. Furthermore, mTOR-dependent inhibition of autophagy promotes proinflammatory signaling via reduced inflammasome clearance [117]. Along these lines it has been recently reported that ethanol exposure (70 mM) activates the NLRP3 inflammasome in induced pluripotent stem cells (iPSCs) and iPSC-derived neural progenitor cells [118]. These results suggest that inflammation, a hallmark of alcohol-mediated tissue damage, may also participate in neurogenesis derangement by ethanol. Taken together, the above cues strongly call for further research in the role of the mTOR cascade, amenable of pharmacological control, in ethanol effects on NSC function and by extension on ethanol-related stem cell failure.

### Effects on nicotinamide adenine dinucleotide

Hepatic ethanol oxidation causes a significant change of the ratio between the oxidized and reduced forms of nicotinamide adenine dinucleotide (NAD^+^/NADH), leading to altered gene expression and epigenetic changes [119, 120]. Interestingly, it has been demonstrated that low levels of NAD^+^ determine a reduction of self-renewal and differentiation capabilities of neural stem cells [121]. Consistently, NAD^+^ repletion has been associated with improved retention of muscular, neural and melanocyte stem cell pools in aged mice [122] and with attenuation of alcohol-associated hepatic lesions [123]. NAD^+^ beneficial effects likely involve several NAD-dependent pathways and effectors, including sirtuins, a class of protein deacylase extensively linked to extended longevity in model organisms and to health span in mammals through antioxidant, prosurvival and metabolism-regulating functions [124]. Interestingly, sirtuins have been identified as potential targets of ethanol in both animal and human models of hepatocellular carcinoma [125, 126]. In conclusion, by causing a reduction of the intracellular NAD^+^ levels ethanol may negatively impact on the preservation of adult stem cells and by extension on tissue regenerative capacity, while pharmacologic treatments aiming at increasing intracellular NAD^+^ content may help to prevent stem-cell functional decline upon alcohol intake.

### Acetaldehyde production and aldehyde dehydrogenases 2 activity

Ethanol metabolism produces acetaldehyde which is genotoxic, interferes with DNA replication, induces DNA damage and formation of DNA adducts [127]. Different, partially redundant, DNA repair mechanisms have been evolved to protect cells from DNA damage. In particular, the Fanconi anemia pathway is involved in the repair of DNA damage induced by acetaldehyde. Concomitant inactivation of the Fanconi anemia DNA repair pathway and the aldehyde catabolism enzyme ALDH2 results in HSC chromosome damage and loss [91, 92, 128]. This observation, originally demonstrated on murine models, was clinically corroborated by the discovery of a higher prevalence of bone-marrow failure in patients affected by Fanconi anemia harboring also a dominant negative polymorphism in the ALDH2 gene which is frequent in the Asian population [129]. Moreover, the expression BRCA1 and BRCA2 tumor suppressors genes, known to contribute to DNA repair [130], has been associated to a protective role against endogenous acetaldehyde toxicity [131]. Consequently, endogenous aldehydes may be implicated in triggering carcinogenesis in BRCA2 mutation carriers [132].

The mitochondrial ALDH2 is the member of the aldehyde dehydrogenase (ALDH) family which provides the main detoxification activity against acetaldehyde. Moreover, ALDH affects HSCs self-renewal via inhibition of the retinoic acid signaling [133]. Noteworthy, elevated ALDH expression has been reported also in tumor-initiating cells, suggesting a functional role of ALDH activity in determining resistance to current cancer therapies and promoting expansion of cancer stem cells [134]. Therefore, aldehyde dehydrogenase activity has a key role in stem-cell self-protection, maintenance and differentiation. Exposure to ethanol is likely to alter ALDH2 expression levels and functions in stem cells as observed in esophageal epithelium [135]; along similar lines of speculation, it is possible that diverse ALDH levels in subpopulations of stem cells may dictate unique vulnerability to alcohol damage, affecting the dynamic of cell proliferation and cell fate.

### Effects on cell membrane and extracellular matrix

Cell membrane-mediated interactions play a pivotal role in regulating stem cell proliferation and differentiation [136]. Ethanol interacts both with the phospholipids and proteins of the plasma membrane, thereby affecting cell structure and function [137]. Furthermore, ROS production associated with alcohol metabolism results in lipid peroxidation, further affecting membrane behavior. In addition, several studies have demonstrated that alcohol deregulates the extracellular matrix (ECM) deposition in different organs including liver, brain, skeletal and cardiac muscle [138–140]. Stem cells rely on mechanical cues from their niche to drive and regulate cell fate [141, 142]. Therefore, by modifying cell membrane fluidity, inducing conformational changes in membrane proteins and altering ECM remodeling alcohol may perturb stem cell maintenance and differentiation.

### Epigenetic mechanisms and microRNAs

Stem-cell differentiation is a strictly orchestrated process involving multiple regulatory pathways. A complex network of soluble factors such as cytokines, hormones and growth factors, regulates stem cell fate [143]. Alcohol intake is known to disrupt cytokine balance and functions, possibly interfering with stem cell proliferation and differentiation [144]. In addition, dynamic epigenetic mechanisms such as histone modifications, changes in DNA methylation, chromatin remodeling, and non-coding RNAs signaling, play a significant role in stem-cell control of self-renewal and differentiation into tissue-specific lineages [145]. Alcohol metabolism has a profound impact on cellular gene expression via modification in the epigenetic machinery [119, 146, 147]. Extensive epigenetic alteration subsequent to alcohol exposure may interfere with the molecular switches needed for the coordinated process of stem cell differentiation. Indeed, perturbation of the epigenetic profile by alcohol exposure leads to alteration of gene expression of specific genes implicated in neuronal stem-cell maintenance and differentiation [148, 149]. Alteration of chromatin structure promoted by prenatal alcohol exposure has been linked to altered neuron and astrocyte differentiation and FASD [27]. In contrast, a recent large cohort study failed to detect any evidence that maternal alcohol consumption during pregnancy is associated with offspring cord blood DNA methylation [150]. Therefore, the role of epigenetic changes and chromatin structure in the context of FASD is far from being fully elucidated.

In addition, broad alcohol‐mediated changes in microRNA (miRNA) and miRNA‐target gene expression have been observed in utero, possibly contributing to the harmful effects on the developing fetus [151]. Consequently, it has been proposed that alcohol-sensitive circulating miRNAs may represent a promising theranostic marker for FASD [152]. Interestingly, alcohol intake can promote defined changes in the content of miRNA enclosed into extracellular vesicles (EVs) [153], which play a pivotal role in intracellular communication. Therefore, EV cargo analysis might represent a sensitive tool for monitoring progression of alcohol addiction disorders [154].

## Conclusions

Emerging evidence for the impact of ethanol on stem and progenitor cells has added a new layer of biological complexity to our understanding of how alcohol misuse affects adult and developing tissues and especially fetal brain. However, the underlying biochemical mechanisms appear manifold, complex and overall incompletely identified. Hopefully, intense research aimed at delineating the pathogenic molecular interactions that link ethanol intake to stem-cell damage will in the next few years provide the immediate benefit of uncovering the processes leading (or at least contributing) to alcohol toxicity. This will be in turn instrumental to develop novel therapeutic strategies to reverse or prevent the effects of alcohol intoxication.

## Abbreviations

ADH: Alcohol dehydrogenase
ALD: Alcoholic liver disease
ALDH: Acetaldehyde dehydrogenase
BM: Bone marrow
CYP2E1: Cytochrome P450 2E1
DG: Dentate gyrus
ESC: Embryonic stem cells
EV: Extracellular vesicles
FASD: Fetal alcohol spectrum disorders
HSC: Hematopoietic stem cells
MSC: Mesenchymal stromal cells
mTOR: Mammalian target of rapamycin
NADH: Nicotinamide adenine dinucleotide
NSC: Neural stem and progenitor cells
PPAR: Peroxisome proliferator-activated receptor
ROS: Reactive oxygen species
